# An Anatomical, Sonographic, and Computed Tomography Study of the Transversus Abdominis Plane Block in Cat Cadavers

**DOI:** 10.3390/ani12192674

**Published:** 2022-10-05

**Authors:** Marta Garbin, Sabrine Marangoni, Cyrielle Finck, Paulo V. Steagall

**Affiliations:** 1 Faculty of Veterinary Medicine, Université de Montréal, Saint-Hyacinthe, QC J2S 2M2, Canada; 2 Department of Veterinary Clinical Sciences and Centre Animal Health and Welfare, Jockey Club College of Veterinary Medicine and Life Sciences, City University of Hong Kong, Hong Kong, China

**Keywords:** analgesia, CT scan, feline, pain management, spread, TAP block, transverse abdominis plane, US-guided, regional anaesthesia

## Abstract

**Simple Summary:**

The *transversus abdominis* plane (TAP) block is a locoregional technique used for postoperative analgesia after abdominal surgery, reducing the consumption of systemic analgesic drugs. The aim of this cadaveric study was to evaluate the spread of an anaesthetic-contrast dye solution administered by an ultrasound-guided 1-point (lateral; TAP-L approach) or 2-point (subcostal and lateral; TAP-SL approach) TAP block technique. Contrast distribution was assessed by computed tomography whereas nerve staining was assessed by anatomical dissection. The 2-point-injection technique provided a wider injectate spread within the TAP than the 1-point technique. Further clinical studies are warranted to investigate the analgesic effect of these TAP block approaches in cats undergoing abdominal surgery.

**Abstract:**

This study compared the distribution of a bupivacaine–iopamidol–dye solution following ultrasound-guided in-plane TAP injection using a 1-point (TAP-L) or 2-point (TAP-SL) approach in cat cadavers. Two cadavers were used to study the TAP sonoanatomy while eight cadavers were enrolled in a randomized, prospective, blinded investigation. Each cat randomly received a TAP-L with 0.5 mL/kg in one hemiabdomen and a TAP-SL with 0.25 mL/kg/point in the contralateral hemiabdomen. After injection, computed tomography and dissection were performed to assess contrast distribution and number of stained target nerves. TAP-SL resulted in a wider contrast spread (mm) compared with TAP-L (87 ± 7 versus 71 ± 9; *p* = 0.002). The prevalence of nerve staining was higher using TAP-SL than TAP-L (*p* = 0.001). The ventral branches of T10, T11, T12, T13, L1 and L2 were stained in 2/8, 2/8, 5/8, 7/8, 4/8 and 1/8, and in 7/8, 7/8, 8/8, 8/8, 8/8 and 1/8 using TAP-L and TAP-SL approaches, respectively. Computed tomography and dissection identified minimal injectate intraperitoneally or within the falciform ligament fat following 1 TAP-L and 2 TAP-SL. Ultrasound-guided TAP-SL provided better injectate distribution around the thoracolumbar spinal nerve branches than TAP-L.

## 1. Introduction

Locoregional anaesthesia is employed in a multimodal analgesic protocol to manage pain, decrease the consumption of systemic analgesic drugs (i.e., opioids) and their potential adverse effects, and potentially decrease the hospital length of stay [1]. The ultrasound (US)-guided *transversus abdominis* plane (TAP) block is a locoregional technique used in humans and animals to provide perioperative analgesia by blocking the sensory component of the nerves supplying the abdominal wall and the underlying parietal peritoneum [2,3,4,5,6,7]. The TAP is the intermuscular fascial plane between the muscle (m.) *transversus abdominis*, the innermost muscle of the abdominal wall, and m. *obliquus internus* or m. *rectus abdominis* and contains the ventral branches of the thoracolumbar (T9–L3) spinal nerves supplying the skin, the abdominal wall and the underlying peritoneum [8]. An adequate volume of local anaesthetic injected into the TAP is expected to provide sensory blockade of the cranial and middle regions of the abdomen. Therefore, the TAP block provides somatic analgesia for surgical procedures involving the abdominal wall (e.g., hernia or laceration repairs, mastectomy, celiotomy [3,4,9]). The veterinary literature is still limited in recommending the TAP block versus other locoregional anaesthetic techniques such as epidural, *quadratus lumborum* block or intraperitoneal administration of local anaesthetics. However, the TAP block does not cause pelvic limb motor impairment or urinary retention that can be observed after epidural administration of local anaesthetics and opioids.

In veterinary medicine, an US-guided TAP block was firstly described in a Canadian lynx as a bilateral 1-point injection of local anaesthetic within the mm. *transversus abdominis* and *obliquus internus abdominis* performed at the midpoint between the last rib and the iliac crest using a lateral approach with a transversal needle orientation [9]. The US-guided TAP block has now been studied in small animals. In dogs, a TAP injection using a single lateral injection failed to stain all target nerves because the injectate solution tended to stay near the injection point, even when large volumes of injectate were used [10,11]. To obtain a wide local anaesthetic spread within the TAP, and consequently an enhanced nerve blockade, multiple-point TAP injections have been used [2,8,12]. Nevertheless, a cadaveric study in dogs revealed that thoracic nerve staining (T9–T12) was rare following a 2-point lateral TAP approach [12]. To target these nerves, a subcostal approach to the TAP has been suggested, which consists of the injection of local anaesthetic in the plane between the mm. *transversus abdominis* and *rectus abdominis* [13]. Recently, an investigation into canine cadavers showed that a combination of subcostal and lateral TAP approaches provides a more consistent staining of the thoracolumbar nerves (T9–L2). However, a gap staining was observed at the level of T13 and it is not known whether needle orientation (longitudinal versus transversal) affects the injectate spread following lateral TAP injection [14]. These studies show that there is no current consensus on the volume of injection (as volumes from 0.3 to 1 mL have been used) or the most appropriate technique (lateral-transversal versus lateral-longitudinal versus subcostal combined with lateral) to perform the TAP block in small animals.

In cats, a recent cadaveric study concluded that a 3-point TAP injection with a combination of subcostal and lateral-transversal TAP approaches is necessary to stain the thoracolumbar spinal nerve branches that provide sensory abdominal innervation [8]. A clinical study showed that a bilateral 1-point lateral-longitudinal TAP block with 1.5 mL of a lidocaine–bupivacaine solution per hemiabdomen (0.3–0.46 mL/kg/point) provided analgesia in cats undergoing ovariectomy [4]. The literature is scarce in cats, and different techniques and volumes of administration are employed in this species. Therefore, anatomical and computed tomography studies may provide further evidence of nerve staining and possible complication of different TAP block techniques. These cadaveric studies may constitute the basis for in vivo future studies involving the pharmacokinetics of local anaesthetics and clinical trials investigating postoperative analgesia following the TAP block in cats.

The objective of this study was to compare the distribution pattern of 0.5 mL/kg of a solution administered by either a 1-point lateral-longitudinal TAP injection (TAP-L approach) or a 2-point subcostal and lateral–longitudinal TAP injection (TAP-SL approach) in cat cadavers using anatomical dissection and computed tomography.

The hypothesis was that TAP-SL approach would result in a wider spread of injectate and staining of thoracolumbar spinal nerve branches compared with the TAP-L approach.

## 2. Materials and Methods

This prospective, randomized, blinded, cadaveric study was approved by the Institutional animal care and use committee (Comité d’éthique de l’utilisation des animaux) of the Université de Montréal (no. 21-Rech-2111).

A total of 10 frozen cadavers of adult cats (eight domestic shorthair, three males and five females; two female domestic longhair) euthanized for reasons unrelated to this study were used. Exclusion criteria included body weight < 2 kg or >6 kg, body condition score > 7 or <3 on a scale from 1 to 9, and any evidence of skeletal and/or muscle injuries. Each cadaver was thawed at room temperature for 48 h prior to the experiment, and the thoracic and abdominal areas were clipped.

The study was divided in two phases: gross anatomy and sonoanatomy study (phase I), and injectate spread evaluation and nerve staining after TAP injections by computed tomography (CT) and dissection (phase II).

### 2.1. Phase I: Anatomical and Ultrasound Study

Two cadavers were used to describe the relevant gross anatomy of the abdominal wall and spinal nerves (T9–L3). Dissections were performed on one hemiabdomen. The skin, the m. *obliquus externus abdominis*, the m. *rectus abdominis*, and the m. *obliquus internus abdominis* were dissected and reflected laterally to expose the nerves innervating the abdominal wall. Subsequently, dissections were performed to identify the thoracolumbar nerve branches up to their origin at the level of the intervertebral foramina. Afterwards, the contralateral hemiabdomen was sonographically evaluated and used to identify ultrasonographic landmarks for the TAP injection. Once the investigators (M.G. and S.M.) became familiar with the sonoanatomy, a 50 mm and 22-gauge spinal needle (BD Spinal Needle; BD Medical, Franklin Lakes, NJ, USA) was used to test an US-guided in-plane TAP subcostal and lateral approach (US settings described below). Two dye solutions, yellow and lime, were prepared using 0.5 mL of permanent tissue marking dye (Davison Marking System; Bradley Products Inc., Minneapolis, MN, USA) diluted in 50 mL of 2% lidocaine (Lurocaine, Vetoquinol, Lavaltrie, QC, Canada) as previously described [15]. A volume of 0.1 mL of the yellow-coloured solution was injected under sonographic visualization (Edge II; Sonosite Inc., Bothell, WA, USA) between the mm. *rectus abdominis* and *transversus abdominis*, and between the mm. *transversus abdominis* and *obliquus internus abdominis*, whereas the same volume of the lime-coloured solution was injected between the mm. *obliquus internus abdominis* and *obliquus externus abdominis* to identify the anatomical landmarks. Finally, the hemiabdomen was dissected to confirm injectate spread.

### 2.2. Phase II: Injectate Spread Evaluation after Ultrasound-Guided TAP Injections

Eight cat cadavers were used to describe the US-guided injection of a bupivacaine–iopamidol–dye solution into the TAP by two approaches and evaluate the injectate spread and nerve staining by CT and dissection.

**Figure 1:**
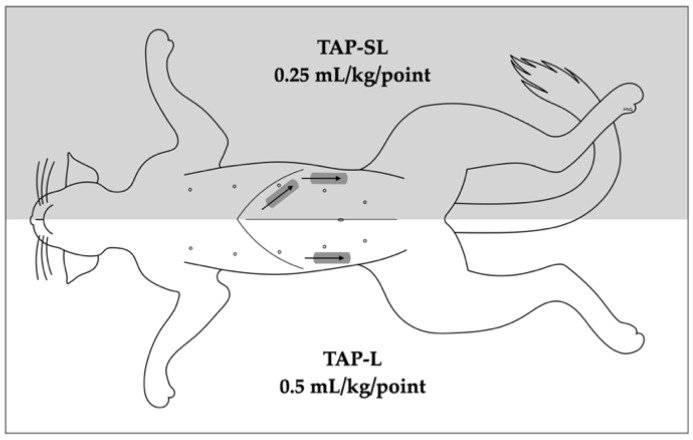
Schematic representation of 1- or 2-point ultrasound-guided *transversus abdominis* plane (TAP) injections. A 1-point TAP injection (lateral; TAP-L approach) was performed in one hemiabdomen, whereas 2-point TAP injection (subcostal and lateral; TAP-SL approach) was performed on the contralateral hemiabdomen. The grey rectangles indicate the transducer position whereas the arrows indicate the needle orientation.

In each cat, a 1-point TAP injection was performed on one hemiabdomen (lateral approach, TAP-L), whereas a 2-point TAP injection was performed on the contralateral hemiabdomen (subcostal and lateral approach, TAP-SL; Figure 1). The side allocation and the TAP block approach were randomly selected by one of the investigators (M.G.) using a randomization order by Microsoft Excel for Mac Version 16.13 (Microsoft Corporation, Redmond, WA, USA).

The TAP-L was performed injecting 0.5 mL/kg of solution, whereas the TAP-SL was performed using the same volume divided in two aliquots of 0.25 mL/kg per injection point (Figure 1). The injectate solution was prepared diluting 0.2 mL of permanent tissue dye (Davison Marking System; Bradley Products Inc., Minneapolis, MN, USA) and 2 mL of iopamidol (Isovue 300, Bracco Imaging Canada, Montréal, QC, Canada) with 20 mL of bupivacaine hydrochloride 0.25% (Bupivacaine Injection BP 0.25%; SteriMax Inc., Oakville, ON, Canada).

#### 2.2.1. Ultrasound-Guided TAP Injection

Sonographic imaging was performed using a 38 mm, 13–6 MHz, linear transducer connected to a portable US machine (Edge II; Sonosite Inc., Bothell, WA, USA). The US setting was adjusted at the minimum depth (1.9 mm) while the gain was adjusted to optimize the image. A 22-gauge, 50 mm Quincke spinal needle (BD spinal needle; Becton Dickinson & Co., Franklin Lakes, NJ, USA) connected by a T-port (Med-RX extension set with t-connector; CHS Ltd., Oakville, ON, Canada) to a prefilled 5 mL syringe, was used to perform the TAP injections. All the injections were performed with the cat in dorsal recumbency by an anaesthesiologist familiar with US-guided TAP blocks (M.G.).

**Figure 2:**
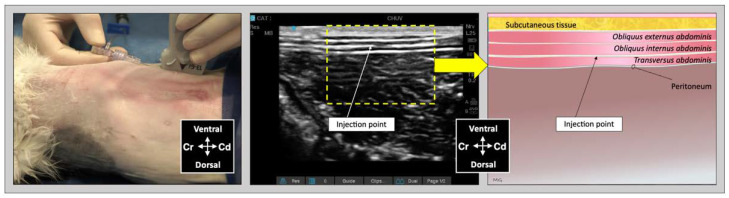
Ultrasound transducer position, needle puncture site, sonographic image, and schematic representation of the lateral TAP injection in a cat cadaver; Cd, caudal; Cr, cranial, L, lateral; Lt, left; M, medial; Rt, right. Adapted from: Garbin, M.; Benito, J.; Ruel, H.L.M.; Watanabe, R.; Monteiro, B.P.; Cagnardi, P.; Steagall, P.V. Pharmacokinetics of bupivacaine following administration by an ultrasound-guided transversus abdominis plane block in cats undergoing ovariohysterectomy. Pharmaceutics 2022, 14, 1548. https://doi.org/10.3390/pharmaceutics14081548 (accessed on 1 August 2022).

For the lateral TAP injection, the transducer was positioned parallel to the long axis of the cat (longitudinal plane), at 3–4 cm lateral to the abdominal midline and about 1 cm lateral to the mammary line, immediately caudal to the last rib (Figure 2). The transducer was slide laterally until the belly of the m. *obliquus internus abdominis* could be identified between the mm. *obliquus externus* and *transversus abdominis*. The needle was introduced in-plane in a ventrocranial-to-dorsocaudal orientation and continually visualized until its tip was advanced into the TAP between the m. *obliquus internus abdominis* and the m. *transversus abdominis* (Figure 2). Then, the assigned injectate volume was administered (0.5 mL/kg in TAP-L versus 0.25 mL/kg in TAP-SL).

**Figure 3:**
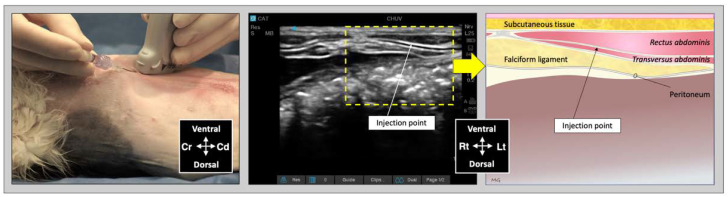
Ultrasound transducer position, needle puncture site, sonographic image, and schematic representation of the subcostal TAP injection in a cat cadaver; Cd, caudal; Cr, cranial, L, lateral; Lt, left; M, medial; Rt, right. Adapted from: Garbin, M.; Benito, J.; Ruel, H.L.M.; Watanabe, R.; Monteiro, B.P.; Cagnardi, P.; Steagall, P.V. Pharmacokinetics of bupivacaine following administration by an ultrasound-guided transversus abdominis plane block in cats undergoing ovariohysterectomy. Pharmaceutics 2022, 14, 1548. https://doi.org/10.3390/pharmaceutics14081548 (accessed on 1 August 2022).

For the subcostal TAP injection, the transducer was initially positioned perpendicular to the long axis of the cat, immediately caudal to the xiphoid process, over the ventral midline. After identification of the *linea alba*, the transducer was slid laterally and parallel to the costal arch and oblique to the midline (Figure 3). The needle was introduced in-plane in a ventromedial-to-dorsolateral orientation and continuously visualized while advanced towards the TAP until its tip was inserted into the fascial plane between the mm. *rectus abdominis* and *transversus abdominis*. Then, an injectate volume of 0.25 mL/kg was administered by TAP-SL.

To improve the sonographic visualization of the needle tip within the fascial planes, the US image was zoomed in proximity of the injection site. Correct positioning of the needle tip was confirmed by visualizing the hydrodissection of the TAP following injection of 0.1 mL injectate solution, prior to the assigned volume. If hydrodissection of the target plane was not observed, the needle was redirected, and the test volume repeated until correct positioning was achieved. The quality of needle visualization was rated as good when both shaft and tip of the needle could be visualized; poor when only the needle tip was clearly visualized; or absent, when the needle tip could not be visualized. US images of each injection were recorded. Complications of the TAP-L and TAP-SL techniques, including needle advancement within the peritoneum or abdominal cavity, or spread of injectate to non-target fascial planes, were assessed and noted.

#### 2.2.2. Computed Tomography Study

Thirty minutes after the last TAP injection, each cadaver underwent a full body CT examination in dorsal recumbency to observe contrast distribution. Scans were obtained using a 16-slice multidetector CT scanner (Hi-speed ZXi; General Electric, Milwaukee, WI, USA). The CT images were obtained in a helical mode at 100 kVp, 300 mA, slice thickness 0.625 mm, and reconstructed using low-pass and high-pass filter algorithms. A board-certified radiologist (C.F.) who was blinded to the treatments provided image interpretation using the Horos™ software (Horosproject.org, Nimble Co LLC d/b/a Purview in Annapolis, MD, USA). The following CT features were recorded: total length (mm) of the contrast spread within the TAP in all directions (cranial-to-caudal, dorsal-to-ventral, and medial-to-lateral), and any intra-abdominal contrast migration (yes/no). The predominant distribution patterns throughout the abdominal musculature for TAP-L and TAP-SL approaches were also described.

#### 2.2.3. Anatomical Dissection

About 30 min after the end of CT examination, the cadavers were dissected to observe dye distribution and thoracolumbar spinal nerve ventral branch staining within the TAP. Two researchers (S.M. and M.G.) performed the dissections and the evaluation of the dye spread. Each cadaver was initially positioned in dorsal recumbency, and the skin was incised along the ventral midline from the mid-thorax to the pubis. The rectus sheath was dissected from the *linea alba*. The aponeurosis of the m. *obliquus externus abdominis* was identified, dissected, and the belly of the muscle was reflected laterally to expose the aponeurosis of the m. *obliquus internus abdominis.* The m. *rectus abdominis* was detached from its lateral margin and dissected medially to expose the m. *transversus abdominis* and its aponeurosis. At this point, the cadaver was placed in lateral recumbency with the hemiabdomen to be dissected uppermost. The aponeurosis of the m. *obliquus internus* was dissected and reflected dorsally to expose the ventromedial branches of the thoracolumbar spinal nerves and the m. *transversus abdominis.*

The ventromedial branches of the spinal nerves T10–L2 were considered the target nerves. Successful staining was defined as circumferential nerve staining with a length ≥ 1 cm [16]. Location and staining of the ventromedial branches of T9 and L3 nerve branches were also noted. The cat was then positioned in the opposite lateral recumbency, and the dissection was repeated in the contralateral hemiabdomen.

After nerve staining evaluation, the cat was repositioned in dorsal recumbency, the abdominal cavity was exposed by incision of the *linea alba,* and the abdominal organs were carefully inspected to assess the presence of the bupivacaine–iopamidol–dye solution. Other complications such as presence of dye intramuscularly or in non-target fascial plane or in the abdominal cavity were recorded.

### 2.3. Statistical Analysis

The number of cats enrolled in the study as well as the total volume of 1 mL/kg of injectate solution per cat, corresponding to 0.5 mL/kg of solution per hemiabdomen, were based on similar cadaveric studies in animals [8,14,15]. Statistical analyses were performed using SPSS Version 28.0 (IBM SPSS Statistic; IBM Corp., Armonk, NY, USA). The Shapiro–Wilk test was used to assess data normality. Data are presented as mean ± standard deviation when normally distributed and as median (range) when not normally distributed. The spread of contrast following TAP-L versus TAP-SL was compared using the Student’s *t* test. The number of ventral nerve branches successfully stained were compared with the Mann–Whitney U test, whereas any difference in the single nerve staining was compared with the Fisher’s exact test. Differences were considered statistically significant when *p* < 0.05.

## 3. Results

### 3.1. Phase I: Description of Gross Anatomy and Sonoanatomy

**Figure 4:**
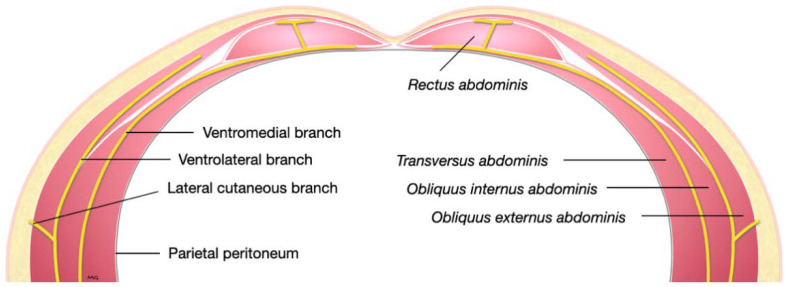
Schematic representation of a transverse section of the lower abdominal wall demonstrating the course of the ventral branches of a lumbar nerve.

**Figure 5:**
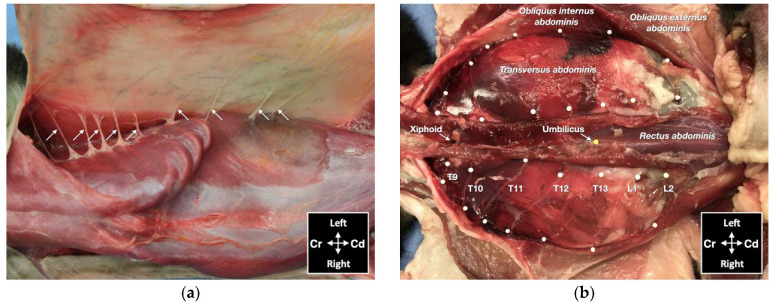
Gross anatomical dissection of two cat cadavers: (**a**) the lateral cutaneous branches of thoracic (T) and lumbar (L) spinal nerves are indicated by arrows; (**b**) the ventromedial branches of the spinal nerves from T9 to L2 are indicated by white pins; Cd, caudal; Cr, cranial.

One male and one female cat weighing 4.1 and 3.7 kg, respectively, were studied. The observed anatomy of the abdominal wall musculature and innervation is shown in Figure 4. The belly of the m. *transversus abdominis* extended through almost the entire abdominal cavity, ended at half of the width of the m. *rectus abdominis* and extended in a thin aponeurosis to the linea alba. The belly of the m. *obliquus internus abdominis*, which was found between m. *transversus abdominis* and the m. *obliquus externus abdominis*, ended at the mid-width of the hemiabdomen (at about 1 cm lateral to the mammary glands line) and extended in a long aponeurosis to the rectus sheath. The m. *obliquus externus abdominis* is found along the lateral and ventral part of the abdomen, ending with the most superficial aponeurotic layer of the rectus sheath. Finally, the m. *rectus abdominis* extended longitudinally in the ventral abdominal wall from the external surface of the thorax to the pubic bone and it is enclosed in the rectus sheath, which multilayered aponeuroses were difficult to dissect. In one cat more than the other, a layer of adipose tissue was observed beneath and immediately caudal the costal arch, between the mm. *rectus abdominis* and *transversus abdominis*, extending laterally between the mm. *obliquus externus* and *internus abdominis*. The ventral branches of the spinal nerves T9–L3 were identified in both cadavers. These branches were further divided into ventrolateral and ventromedial branches. The ventrolateral branches were located at half of the width of thorax and abdomen (Figure 5a). These branches are found between the intercostal muscles or between the mm. *obliquus internus abdominis* and *obliquus externus abdominis*, before they end at the subcutaneous and cutaneous tissues. The ventromedial branches were located within the TAP, with different endings: the T9 branch at the lateral margin of the xiphoid process, T10–L2 at the ventral midline (with T12 and T13 being cranial and caudal to the umbilicus, respectively) and further divided into terminal branches in the belly of m. *rectus abdominis*, and L3 in the belly of the m. *transversus abdominis* at the level of the pubis (Figure 5b).

**Figure 6:**
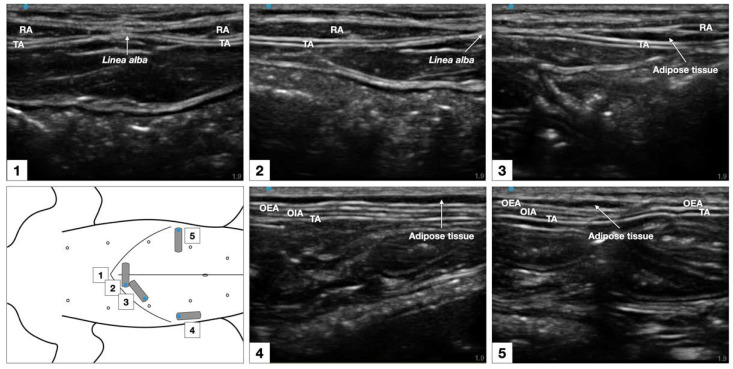
Sonographic identification of the TAP in a cat cadaver. At the subcostal region, the m. *transversus abdominis* (TA) was visualized as the more hypoechoic muscle layer just beneath the m. *rectus abdominis* (RA), and the TAP was discerned as a hyperechoic line between the two muscles. At the lateral region of the abdomen (at mid-level between axilla and iliac crest, and lateral to the mammary gland line), three hypoechoic layers were observed, corresponding to m. *transversus abdominis*, m. *obliquus internus abdominis* (OIA) and *obliquus externus abdominis* (OEA) from a deep to superficial order. The TAP was identified as a hyperechoic line superficial to the m. *transversus abdominis*. At the abdominal level, 1–3 cm lateral to the ventral midline (between the lateral margin of the m. *rectus abdominis* and the medial margin of the m. *obliquus internus abdominis*) the TAP and the aponeurosis of the m. *obliquus internus abdominis* appeared as a unique tick hyperechoic line.

The sonographic appearance of the TAP varied depending on the position of the US transducer and is shown in Figure 6.

### 3.2. Phase II: Injectate Distribution Study by Ultrasonography, Computed Tomography and Dissection

Two male and six female adult cats were used. Median (range) body weight and body condition score were 3.0 (2.3–4.4) kg and 3.5 (2–5) in a scale from 1 to 9, respectively.

#### 3.2.1. Ultrasound-Guided TAP Injection

A total of eight lateral injections by the TAP-L approach and eight lateral and eight subcostal injections by the TAP-SL approach were performed. Needle visualization was scored as “good” in all subcostal injections of the TAP-SL, “poor” in six TAP-L and six TAP-SL, and never “absent”.

Spread of injectate was observed exclusively in the target plane following eight TAP-L and six TAP-SL. Following two subcostal injections in the TAP-SL approach, injectate spread was observed both dorsal and ventral to the m. *transversus abdominis*. No intra-abdominal cavitary spread was observed with either approach.

#### 3.2.2. Computed Tomography

**Figure 7:**
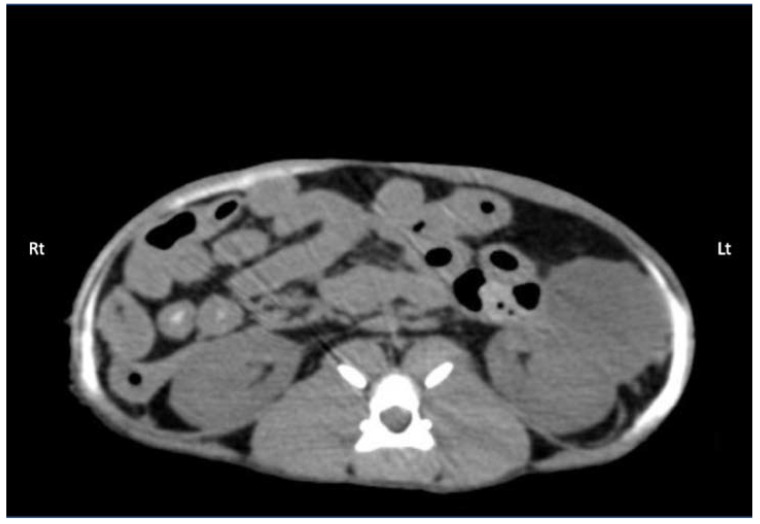
Computed tomography (CT) image showing the distribution of 0.5 mL/kg bupivacaine–iopamidol–dye solution administered by two approaches of the TAP injection in a cat; a TAP-L injection was performed in the left (Lt) hemiabdomen and a TAP-SL in the right (Rt) hemiabdomen; WL 50, WW 350. Ventral is at the top of the image.

**Figure 8:**
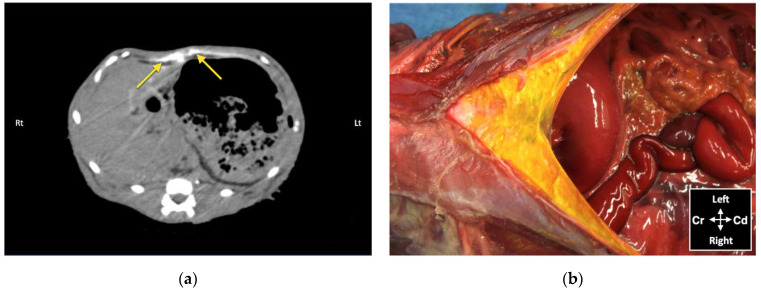
Bupivacaine–iopamidol–dye distribution in the falciform ligament following subcostal TAP injection (TAP-SL approach) in a cat cadaver: (**a**) Transverse CT image showing injectate distribution ventral to the m. *transversus abdominis* and dorsal to the liver and the stomach (yellow arrows); WL 50, WW 350. Right (Rt) is to the left (Lt) of the image and ventral is at the top of the image; (**b**) Anatomical dissection of the same cat showing injectate distribution in the ligament after incision of skin, subcutaneous tissue and *linea alba*; Cd, caudal; Cr, cranial.

Contrast solution was identified in the target fascial plane in eight TAP-L and eight TAP-SL (Figure 7). Following all 16 lateral TAP injections, the contrast solution distribution was assessed as “possible partial intramuscular infiltration in the m. *transversus abdominis*” and following 7 out of 16 lateral TAP injections (3 TAP-L and 4 TAP-SL) as “possible focal spread between the mm. *obliquus internus* and *externus abdominis*”. Focal (1.5 mm) intraperitoneal contrast solution was observed following two lateral TAP injections (1 TAP-L and 1 TAP-SL). In 2 TAP-SL, contrast solution was observed within the falciform ligament fat (Figure 8a). Contrast solution was not observed in the abdominal cavity or organs.

The TAP-SL resulted in a significant wider cranial-to-caudal spread of contrast (87 ± 7 mm) compared with the TAP-L (71 ± 9 mm; *p* = 0.002). No difference was found in the dorsal-to-ventral or medial-to-lateral spread of injectate between the two TAP approaches (Table 1).

#### 3.2.3. Anatomical Dissection

**Figure 9:**
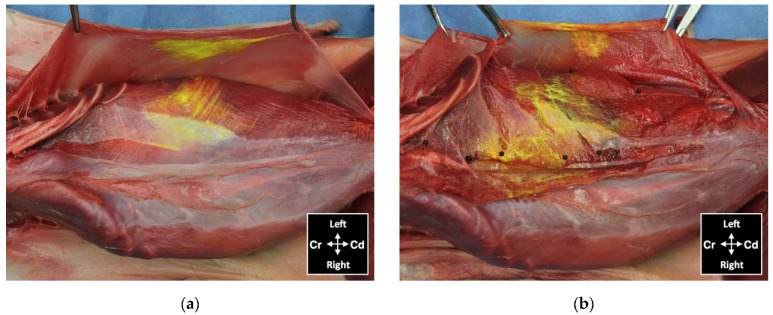
View of the abdominal wall after skin and subcutaneous tissue removal showing distribution of dye administered by an ultrasound-guided 2-point subcostal and lateral TAP injection (TAP-SL approach) in a cat cadaver: (**a**) minor staining of the m. *obliquus externus abdominis*, held with forceps, and in the TAP ventral to the m. *obliquus internus abdominis*; (**b**) ventromedial branches of thoracic and lumbar nerves localized in the TAP (black pins) and stained by the injected solution; Cd, caudal; Cr, cranial.

Dissection of the abdominal wall showed that all injections were performed on the target fascial plane, either between the m. *transversus abdominis* and the m. *rectus abdominis* for the subcostal injections or between the m. *transversus abdominis* and the m. *obliquus internus abdominis* for the lateral injections. Minimal dye solution was found in a fascial plane superficial to the TAP, between the mm. *obliquus internus abdominis* and *obliquus externus abdominis* or between their aponeuroses, in all 16 lateral injections (Figure 9). The surface of these muscles was coloured by the injectate with higher intensity in the eight TAP-L than in the eight TAP-SL. In two cadavers, the fat of the falciform ligament was stained in one and both hemiabdomens (the dye solution crossing the ventral midline) after subcostal TAP injections (Figure 8b).

**Figure 10:**
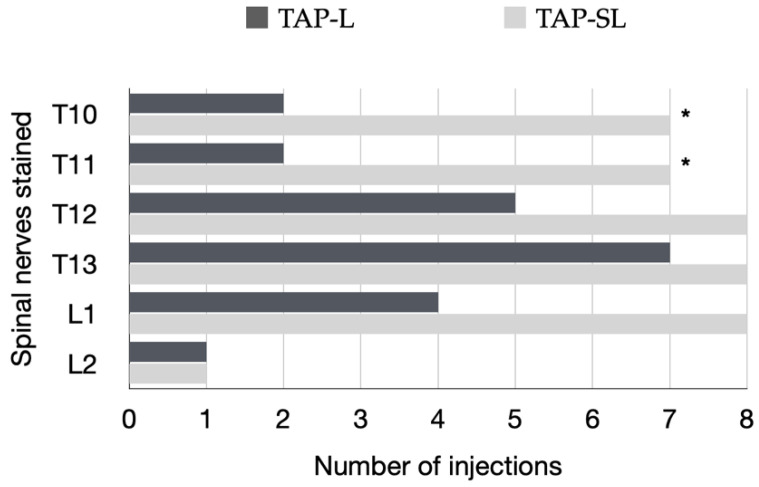
Specific ventromedial branches of the thoracolumbar nerves (T10–L2) per hemiabdomen stained for a length ≥ 1 cm with either a 1-point TAP injection (lateral; TAP-L approach) or a 2-point TAP injection (subcostal and lateral; TAP-SL approach) in eight cat cadavers. A total of eight TAP-L and eight TAP-SL injections were performed; * *p* = 0.04.

In all cadavers, the ventromedial branches of the T10–L3 spinal nerves were identified within the TAP. The ventromedial branch of T9 was identified within the TAP in 12 out of 16 hemiabdomens at the level of the xiphoid process. The ventromedial branch of L3 reached the ventral midline at the level of the pubis in one cat. The ventromedial branch of the T10–L2 spinal nerves successfully stained by TAP-L and TAP-SL is summarized in Figure 10. The TAP-L and TAP-SL approaches stained three (0–4) and five (4–5) out of six target thoracolumbar nerves per hemiabdomen, respectively (*p* = 0.001). The total number of target nerves stained with the TAP-SL approach was 39 out of 48 (81.3%) and was significantly higher than the number of target nerves stained with the TAP-L approach, which was 21 out of 48 (43.8%). When analysing individual nerve staining by both approaches, the TAP-SL resulted in a significantly higher success rate of staining T10 and T11 compared with the TAP-L (*p* = 0.04). No significant difference was observed between the two approaches in staining of nerves caudal to T12. The nerve L2 was stained only in one out of eight injections by either approach. The ventromedial branches of T9 and L3 were not stained by any injection.

## 4. Discussion

This study described the anatomical and sonographic findings of the TAP in cats. Injectate distribution and consistency of nerve staining was also assessed by ultrasonography, CT and dissection after a TAP injection using two approaches. This information allows better understanding of potential local anaesthetic distribution, complications and limitations of this locoregional technique in the clinical setting.

In this study, a significant difference in cranial-to-caudal injectate spread was found between the 1-point TAP-L approach and the 2-point TAP-SL approach. Nevertheless, the most cranial and caudal margins of the solution spread corresponded to the nerve T10 and L2, with either approach. On the other hand, the TAP-SL approach resulted in a more consistent staining of the target nerves than TAP-L, which could represent a more predictable distribution of an anaesthetic solution in cats.

The ventromedial branch of L2 was stained only in 12.5% of cases after TAP-SL and TAP-L. Therefore, the caudal portion of the abdominal wall could possibly not be desensitized, depending on the incision size, by a local anaesthetic. The lateral TAP injection was performed inserting the needle immediately caudal to the last ribs at 3–4 cm from the ventral midline; it is possible that a more caudal injection point (about 1–1.5 cm from the caudal margin of the last rib—in proximity of the umbilicus) would lead to a better caudal injectate distribution and consistent staining of L2 than the present technique. Further anatomical studies are warranted to confirm or reject this hypothesis.

The ventromedial branch of T10, T11, T12, T13, L1 and L2 was stained in 87.5%, 87.5%, 100%, 100%, 100% and 12.5% of cases with our lateral-longitudinal TAP-SL approach when compared with 57.1%, 100%, 87.5%, 28.5%, 42.8% and 87.5%, respectively, by a previously reported lateral-transversal TAP-SL approach [8]. This shows that the needle orientation influences nerve staining after a lateral TAP injection, as the lateral-longitudinal TAP injection resulted in a more consistent nerve staining from T10 to L1 than the lateral-transversal TAP injection. However, the lateral-transversal TAP-SL resulted in a consistent staining of L2. This previous study obtained more consistent staining of L2 branches than with the 2-point lateral-transversal TAP-SL technique when the same injectate volume was divided in three aliquots and injected using a 3-point lateral-transversal TAP-SL technique [8]. At this point, a clinical trial is necessary to assess which TAP injection technique would be more effective (i.e., a 2-point TAP-SL with a lateral-longitudinal approach or a 3-point TAP-SL with a lateral-transversal approach).

Individual variability in the pathway of the thoracolumbar nerve branches in the TAP was observed as reported in similar cadaveric studies in cats [8] and in dogs [17]. T9 branches were identified within the TAP in 75% of our cats versus 56.2% in a previous study [8]. L3 branches were observed within the TAP in all hemiabdomens. However, in 87.5% of cadavers, it ended within the belly of the m. *transversus abdominis*, whereas in one out of eight cadavers, it reached the midline at the level of the pubis. Neither of the TAP approaches employed in this study stained the ventral branches of T9 and L3. These findings demonstrate that the efficacy of a TAP block may vary due to anatomical individual differences independently of similar volumes of administration and techniques. It is possible that the efficacy of a TAP block may also vary according to the extent (size) and location (cranial versus caudal) of a laparotomy.

In this study, the ventrolateral branches of the thoracolumbar nerves responsible for the abdominal cutaneous dermatome innervation were also stained. This phenomenon was also observed in a previous study [8]; however, the clinical relevance of this finding is unknown. Although the TAP injections were all performed in the target fascial plane, partial intramuscular contrast spread in the m. *transversus abdominis* and suspected presence of contrast between the mm. *obliquus internus* and *externus abdominis* were observed in the CT. During gross anatomical dissection, the m. *obliquus internus abdominis* and sometimes the m. *obliquus externus abdominis* were partially stained by the dye solution, as previously reported [8]. Diffusion of injectate through the thin abdominal musculature may commonly occur after a TAP block in cats without any clinical significance.

Although no bupivacaine–iopamidol–dye solution was observed free in the abdominal cavity, a total of 4 out of 24 injections (16.7%) resulted in intraperitoneal spread of injectate solution. Both CT and dissection revealed the presence of injectate solution into the falciform ligament following two subcostal injections. In these two cases, distribution of injectate solution both in the TAP and ventral to the m. *transversus abdominis* was observed with the US. Nevertheless, gentle repositioning of the needle tip under US visualization resulted in partial intramuscular injection in the m. *rectus abdominis*, suggesting that the bevel of the needle was larger than the TAP and the m. *transversus abdominis*. Analysis of CT images also revealed focal intraperitoneal injections that were not observed with real-time US visualization and could not be identified by gross dissection following two lateral TAP injections. These two focal infiltrations of contrast solution could be explained by puncture of the peritoneum with the needle tip, without evident needle perforation of the abdominal cavity. The Quincke needle used in this study has a 20-degree-long cutting bevel, and the m. *transversus abdominis* of the three cats, in which these complications were observed, was about 0.2 mm thick. It is possible that a different type of needle with a shorter bevel (e.g., an insulated needle for peripheral nerve block or a Touhy epidural needle with a rounder tip) would be more appropriate to perform TAP injections in cats.

The prevalence of intraperitoneal injection reported herein (16.7%) was lower than that observed in similar investigations into dog (23%) and human cadavers (25%) using CT studies [11,18]. Therefore, CT might be a more sensitive method to assess contrast misplacement and to identify intra-abdominal injections than gross anatomical dissection. However, the use of CT to assess the injectate spread before a clinical procedure is not feasible. Hence, particular attention should be taken when performing an US-guided TAP block in small patients, because intraperitoneal injection is a possible risk. In this case, there could be a risk of organ perforation or partial anaesthetic block with this technique.

The injectate volume used was based on a previous cadaveric study in cats [8]. Moreover, a volume of 1 mL/kg of anaesthetic solution is considered a reasonable clinical applicable volume and easily divided in four aliquots. The anaesthetic solution should be diluted before administration without exceeding recommended doses of local anaesthetic. Our group published a recent pharmacokinetic study on bupivacaine administered by TAP block in healthy cats showing that a dose of 2.5 mg/kg is safe [19]; this dose is comparably higher than previously recommended in cats [20], allowing high volumes of administration. A cadaveric study in dogs assessing different volumes from 0.5 to 1 mL/kg, administered by 1-point lateral-transversal TAP block, showed a correlation between volume and number of stained nerves [21]. However, two other cadaveric studies in dogs showed that volumes higher than 0.5 mL/kg/point tend to be pooled in the injection site rather than distributed cranio-caudally along the TAP [10,11].

The findings obtained by cadaveric studies should be carefully applied to a clinical setting. The physical properties of the bupivacaine–iopamidol–dye solution employed in this investigation differ from local anaesthetics, and it is possible that this might have influenced the results. The contrast was diluted to a proportion of 1:10 as previously described in cadaveric studies in humans and dogs [11,18]. A further dilution of the contrast might have reduced the quality of the CT images and the spread analysis. On the other hand, this cadaver study was able to identify potential complications and limitations of this technique in the clinical setting. Indeed, this study allowed us to later perform a pharmacokinetic study [19] of bupivacaine in cats undergoing ovariohysterectomy in which we demonstrated that the TAP-SL is safe and bupivacaine concentrations (2 or 2.5 mg/kg) were below toxic levels. Our group is now conducting a prospective, randomised, masked clinical trial investigating the analgesic efficacy of bupivacaine following a TAP block in cats.

This investigation has limitations, and some have already been discussed. The injectate distribution in a cadaver model may differ from the distribution encountered in live animals because of tissue modifications related to freezing and thawing, systemic drug absorption according to the tissue temperature, and changes in abdominal pressure with breathing. A further limitation is that one researcher performing the dissections was not blinded, which may have biased the interpretation of results. Finally, it is unknown how the results and potential complications would have been changed if the TAP block had been performed by an inexperienced veterinarian.

## 5. Conclusions

The 2-point TAP-SL approach resulted in better injectate distribution than the 1-point TAP-L approach. Although the spinal nerve L2 was rarely stained by either approach, the 2-point TAP injections consistently stained all ventral branches of spinal nerves T10–L1, suggesting that the TAP-SL approach could be superior to the TAP-L approach.

Investigation of the clinical efficacy of an anaesthetic solution injected by a combined subcostal and lateral-longitudinal TAP approach in cats is currently ongoing.

## Figures and Tables

**Table 1:** Mean ± standard deviation of the spread (mm) of a bupivacaine–iopamidol–dye solution injected by a lateral TAP approach (TAP-L) and a subcostal-lateral TAP approach (TAP-SL), assessed via computed tomographic imaging.

Injectate Spread	TAP-L	TAP-SL	*p* Value ^1^
Cranial-to-caudal (mm)	71 ± 9	87 ± 7	0.002
Ventral-to-dorsal (mm)	55 ± 8	58 ± 18	0.64
Medial-to-lateral (mm)	4 ± 1	3 ± 1	0.21

^1^ *p* < 0.05 set for statistical significance.

## Data Availability

Data are available from authors upon reasonable request.
